# An analysis of case studies for advancing photovoltaic power forecasting through multi-scale fusion techniques

**DOI:** 10.1038/s41598-024-57398-z

**Published:** 2024-03-20

**Authors:** Mawloud Guermoui, Amor Fezzani, Zaiani Mohamed, Abdelaziz Rabehi, Khaled Ferkous, Nadjem Bailek, Sabrina Bouallit, Abdelkader Riche, Mohit Bajaj, Shir Ahmad Dost Mohammadi, Enas Ali, Sherif S. M. Ghoneim

**Affiliations:** 1 Centre de Développement des Energies Renouvelables, CDER, Unité de Recherche Appliquée en Energies Renouvelables, URAER, 47133 Ghardaïa, Algeria; 2 Telecommunications and Smart Systems Laboratory, University of ZianeAchour, 17000 Djelfa, Algeria; 3 Materials, Energy Systems Technology and Environment Laboratory, Ghardaia University, Ghardaia, Algeria; 4 Energies and Materials Research Laboratory, Faculty of Sciences and Technology, University of Tamanghasset, Tamanghasset, Algeria; 5 University of Sciences and Technology Houari Boumediene, Alger, Algeria; 6 Department of Electrical Engineering, Graphic Era (Deemed to Be University), Dehradun, 248002 India; 7 Hourani Center for Applied Scientific Research, Al-Ahliyya Amman University, Amman, Jordan; 8 Graphic Era Hill University, Dehradun, 248002 India; 9 Applied Science Research Center, Applied Science Private University, Amman, 11937 Jordan; 10 Department of Electrical and Electronics, Faculty of Engineering, Alberoni University, Kapisa, Afghanistan; 11 Centre of Research Impact and Outcome, Chitkara University Institute of Engineering and Technology, Chitkara University, Rajpura, Punjab 140401 India; 12 Department of Electrical Engineering, College of Engineering, Taif University, P.O. BOX 11099, 21944 Taif, Saudi Arabia

**Keywords:** Time series forecasting, Photovoltaic, Deep learning, Hybrid methods, Signal processing, Energy science and technology, Engineering, Mathematics and computing

## Abstract

Integration renewable energy sources into current power generation systems necessitates accurate forecasting to optimize and preserve supply–demand restrictions in the electrical grids. Due to the highly random nature of environmental conditions, accurate prediction of PV power has limitations, particularly on long and short periods. Thus, this research provides a new hybrid model for forecasting short PV power based on the fusing of multi-frequency information of different decomposition techniques that will allow a forecaster to provide reliable forecasts. We evaluate and provide insights into the performance of five multi-scale decomposition algorithms combined with a deep convolution neural network (CNN). Additionally, we compare the suggested combination approach's performance to that of existing forecast models. An exhaustive assessment is carried out using three grid-connected PV power plants in Algeria with a total installed capacity of 73.1 MW. The developed fusing strategy displayed an outstanding forecasting performance. The comparative analysis of the proposed combination method with the stand-alone forecast model and other hybridization techniques proves its superiority in terms of forecasting precision, with an RMSE varying in the range of [0.454–1.54] for the three studied PV stations.

## Introduction

Solar photovoltaic (PV) energy has recently emerged as a viable alternative to conventional forms of electrical power production, such as fossil fuels^1^ power production. There has been a significant increase in the amount of PV capacity that has been installed throughout the world because solar energy is clean, economical, renewable, and beneficial to the environment. Therefore, photovoltaic (PV) systems provide an efficient alternative to supply distant locations by power, pumping water, and according to grid-connected PV plants, reducing electricity expenses.

PV power production is very sensitive to variations in solar irradiation as well as other factors of the local environment, in contrast to the traditional sources, where electrical power production can be simply managed. As a result, the integration of PV electricity into the electrical grid is a major challenge. Renewable power producers, like other energy producers, should respect the expected electrical power for the market session^2^. Precise PV power assessment is vital for (1) integrating PV power into the grid, (2) selling PV power in the electricity markets, and (3) planning PV plant maintenance.

The most current research on PV power forecasting includes a large number of different forecasting approaches, the choice of which is dependent on the adopted time horizon of the forecast, the utilized inputs, and the forecasting strategy that is chosen. The PV power forecasting techniques may be classified into^3^: (1) physical methods, relying on satellite/sky imagery or numerical weather prediction (NWP) that need post-processing to transform their output to PV power, and (2) data-driven approaches that relate the output of the PV plant to external factors^4^. In the first category, the modeling process necessitates the collection of a substantial amount of data and the execution of several complicated analyses to estimate the parameters of the physical model, which restricts its applicability to the real world. On the other hand, data-driven approaches are extensively utilized in PV generation forecasts due to the fast growth of data mining, machine learning, and deep learning methods in recent years.

In a mathematical sense, the data-driven forecasting category may be classified into three sub-class: linear, non-linear, and hybrid models. The linear models are the simplest kind of these classes, and the most well-known linear forecasting models include the auto-regression (AR), auto-regressive integrated moving average (ARIMA)^5^, and auto-regressive moving average extrapolation (ARMAX)^6^. For one-step forecasting tasks, previous research has revealed that linear forecasting models may usually provide reliable outcomes. Despite this, it is apparent that the PV generation series is not linear. Thus, the majority of academics are concentrating their efforts on the creation of non-linear forecasting models such as artificial neural networks (ANN). Artificial Neural Networks (ANNs) have been shown in prior studies to be an effective tool for accurate predicting. It has been shown that employing ANNs to simulate linear trends may provide mixed results and it is not advised to use ANNs naively in any form of data^7^. Extreme learning machines, fuzzy systems, k-nearest neighbor, support vector machines, and their hybridization have dominated current PV forecasting literature. The behavior of a single model does not result in a more precise forecast of the amount of power produced by PV in a variety of scenarios. One possible explanation for this issue is that the stand-alone approach has certain shortcomings. In such situations, the ideal solution is a hybrid model, which combines two or more methodologies. These sorts of the model have been utilized for numerous forecasting applications to reach improved accuracy. One of the key goals of these models is to study the various combinations of different topologies in enhancing prediction accuracy. By using the advantages of each topology separately, hybrid models may boost forecasting performance. Solving the PV power forecasting issue using hybrid models has shown excellent performance compared with stand-alone models. Hybrid methods for time series forecasting have recently developed as a dynamic and active research area^8^ using different techniques such as metaheuristic algorithms^9^, sparse representation theory^10–12^ and decomposition ensemble learning approaches^13^. A review of various forecasting techniques for solar irradiation and PV power is provided in^13,14^. A trending technique in the field of PV and other short-term forecasting domains is the use of deep learning techniques. Deep learning techniques are a complex and advanced type of machine learning approach. It can derive deep information from the PV power time series and produce high predictive results than other conventional models^15^.

Lulu Wen et al.^16^ developed a DL technique for solar power forecasting on an hourly scale. The forecasting outcomes prove that the DL model provides high precision than MLP and SVM models. A combined variational autoencoder VAE with a deep LSTM model provides low forecasting error (RMSE = 5.471) for short-term forecasting of PV power on different PV systems compared with different machine learning techniques^17^. The LSTM neural network model was proposed in^18^, for daily forecasting PV power. The outcomes of the developed model exhibit high forecasting performance compared to MLP, LR, and persistence methods. The proposed model present also 42.9% RMSE skill compared to the benchmarking models for 1-year testing data. Narvaez et al.^19^ used deep learning techniques for daily and weekly scale forecasting of PV power. The obtained results showed that the DL model performed 38% better than the traditional forecasting method. The integration of the CNN model with LSTM^20^ shows excellent forecasting accuracy compared to other methods for the hourly scale of PV power forecasting. The developed mechanism has yielded the lowest forecasting error in terms of MAE, MAPE, RMSE, 0.0506,13.42, and, and 0.0987, respectively.

Zhen et al. proposed a new combination methodology based on the BI-LSTM model with a genetic algorithm (GA) to improve PV power forecasting^21^. Abdel-Basset et al.^22^, introduce a new learning model PV-Net for short-term forecasting of PV power by reconfiguring the gates of the GRU model utilizing convolution layers. The achieved results show that the proposed PV-Net can extract hidden features from historical PV data and provide high forecasting accuracy. Wang et al.^23^ propose the use of the new deep learning model generative adversarial model networks (GANs) for weather classification employing Wasserstein GAN with gradient penalty and deep CNN-model. Research findings demonstrate the proposed WGANGP offers enhanced precision compared to reference models. Bendaoud et al.^24^ developed a conditional generative adversarial model network (CGAN) for short-term PV power forecasting using exogenous data. Huang et al.^25^, proposed a new combined model for hourly PV power forecasting. In their work, they developed a new time series conditional generative adversarial model TSF-CGAN, the proposed model is built by CNN and the Bi-LSTM model. The proposed combination methodology proves its performance against stand-alone models such as SVM, LSTM, RNN, and MLP models. Recent research has shown that the decomposition-learning strategy enhances the forecasting performance of stand-alone models significantly, and several time–frequency methodologies have been introduced for non-stationary signals analysis.

Wavelet Transform (WT)^26^, Empirical Mode Decomposition (EMD)^27^, Ensemble Empirical Mode Decomposition (EEMD)^28^, Multivariate Empirical Mode Decomposition (MEMD)^29^, Ensemble Empirical Mode Decomposition (EEMD)^30^, a modified variant of the conventional EMD (CEEMDAN)^31,32^ and Iterative Filtering decomposition method (IF)^2^. In this paper, five different decomposition learning approaches were independently investigated before being combined for short-term PV power forecasting, using one dimensional CNN model as an essence regressor owing to its capacity to extract more relevant features from the supplied input data.

The remaining parts of the work are structured as described below. In the second section, a detailed literary analysis of the different decomposition ensemble learning algorithms for PV power forecasting is presented. "Contributions of the paper" section presents the paper's contribution. Case studies, data collection, are presented in "Material and monitoring" section. The fundamental structure of the proposed model is presented in "Methodology" section. "Performance metrics" and "Results and discussion" sections discuss the model assessment and the key findings of this study, respectively.

## Literature review

Scientists have conducted various research on sun radiation evaluation so far, with interesting results. In this section, hybrid decomposition approaches are classified according to the used decomposition method:

### Empirical mode decomposition

Shang and Wei^33^ proposed a multi-stage PV power forecasting model in four regions in USE. They proposed an enhanced version of empirical model decomposition EEMD combined with the SVM regression model for 15-min and daily solar radiation forecasting. Maximize Relevancy Minimize Global Redundancy MRMGR feature selection technique is employed to reduce the huge numbers of decomposed components of endogenous and exogenous in different sites (400–600 feature vectors), after selecting the best candidate feature sets the improved SVM model is coupled with PSO technique for hyper-parameters tuning. The provided results show high forecasting accuracy compared with benchmarking models (MLP, RBF, ANFIS, NNPSO, -MLP, WT-RBF, WT-ANFIS, and WT-PSO).

Eseye et al.,^34^ suggested the use of wavelet transform coupled with POS and SVM models for multi-hour PV power forecasting using previous PV power with SCADA records and physical data. Behera et al.^35^, suggested a new hybrid model for short-term PV power forecasting. The developed model consists of three main steps: firstly, the historical PV power is split into a multi-frequency band using the EMD algorithm. The decomposed component derived from EMD is then fed into the ELM model to generate the specified PV power. The sine cosine algorithm (SCA) is utilized for ELM hyperparameter tuning. The proposed EMD-SCA-ELM method is compared to its counterparts models, SCA-ELM, EMD-ELM, and stand-alone ELM model. The comparison in term of statistical metrics shows that the suggested appraoch provides better performance for multistep forecasting.

Wang et al.^36^, proposed the use of ICEEMDAN decomposition techniques with a modified version of particular swarm optimization (MPSO) for hyper-parameter selection of the SVM model. The suggested strategy is more suitable for other common approaches for PV power forecasting.

Wang et al.^37^, proposed the combination of the variable-weight combination model with ensemble empirical mode decomposition (EEMD) for PV power forecasting. Firstly, EEMD is employed for decomposing PV power data into different sets of frequency ranges. The decomposed components are classified into three main categories, high-frequency, and intermediate-frequency. These three categories are separately forecasted using a parameters-weight integration model and summed to get the actual PV signal. Experimental results show that an individual forecast strategy provides better precision than a direct forecasting method.

Zhou et al.^38^, proposed a new integrated model for PV power forecasting, the developed model was validated on three different databases in Safi-Morocco. The combined model consists of using the CEEMDAN algorithm, multi-objective chameleon swarm algorithm (MOcsa), and four ML and DL models. The CEEMDAN is utilized for PV power decomposition, and the MOcsa strategy is employed for determining the weight coefficient of the used subsystems (Enn, LStm, Cnn, and BiLStm). The proposed forecasting strategy is validated on three data sets with different panel technology (May 25 to July 15, 2018, 5 min time scale) including one polycrystalline (p-Si), one amorphous (a-Si) technique, and eight monocrystalline (m-Si), and proves its prediction performance against different deep learning and machine learning technique in terms of mean relative error (MRE), mean absolute error (MAE), and Symmetric mean absolute percentage error (SMAPE).

Lin et al.^39^ developed a new cascade decomposition using EEMD and VMD algorithms, in which EEMD is used firstly to decompose the given time series into a set of intrinsic mode functions, then VMD is employed to split the first component IMF1 from the EEMD method to obtain more stable components. All the obtained IMFs components are then fed into the BILSTM model for PV power forecasting. The developed approach proves its performance compared to other forecasting models (EEMD-BILSTM, EEMD-LSTM, ANN, LSTM, VMD-GRU, EEMD-ANN, and BILSTM) models.

Niu et al.^40^, proposed multi-stages for short PV power forecasting. In the first stage, the random forest algorithm RF is used to rank the most important exogenous input and then remove the irrelevant data, then the selected factors are moved as weighted values to the improved grey (IGIVA) to select days with identical weather patterns. Time series PV power is decomposed using complementary ensemble empirical mode decomposition (CEEMD). In the modeling stage, the backpropagation neural network (BPNN) is used to forecast the desired output. The proposed RF-CEEMD-DIFPSO-BPNN model shows its forecasting performance and its stability under different weather conditions compared to other studied models.

Zhang et al.^41^, proposed a new integrated model for PV power forecasting which include time series decomposition using improved empirical mode decomposition (IEMD), feature selection strategy using Maximize Relevancy-Minimize Global Redundancy, then PSO–SVM as a forecasting model in which PSO algorithm serves for hyper-parameters selection.

### Wavelet decomposition

Random Vector Functional Link (RVFL)—Seasonal Autoregressive Integrated Moving Average (SARIMA)—model combined with Maximum Overlap DWT was proposed in^42^, for three steps-ahead PV power forecasting in IIT Gandhinagar, India. In their work, the DWT was used to split the PV data into 5 sub-series for each decomposed component both forecasting models were applied, then a convex combination was utilized for the final forecast. The combination of these two models with DWT delivers excellent precision compared to the single models, and the combination of DWT-REVL, DWT-SARIMA.

De Giorgi et al.^43^ proposed the use of wavelet transform WT with least square support vector machine LS-SVM for hourly PV power forecasting 24 h ahead in Italy. In their work, they conducted many experiments and observed that the pairing of LS-SVM and ANN with WT delivers high performance, and for long time horizons WT-LS-SVM reaches the highest performance.

Wan et al.^44^ proposed the use of wavelet transform WT coupled with a deep CNN model to build a WT-DCNN forecasting model for hourly PV power in Belgium. In their work, WT is used to split the PV data into a set of different frequency ranges. The framework consists of a 1D-to a-to-2D-Image layer, convolution layer, pooling layer, 2D-to a 1D-layer, and finally logistic regression layer for each decomposition. Then a wavelet reconstruction phase is employed for the final forecast of the PV power signal. The achieved results show that the hybridization strategy performs better than the stand-alone (SVM and MLP) model and the hybrid WT-SVM in terms of forecasting criteria for all forecasting horizons in the two studied regions.

Chen et al.^45^, proposed a new fusing strategy for PV power forecasting using endogenous data on an hourly scale. In their work, the DWT algorithm was employed with an adaptive neuro-fuzzy inference system (ANFIS). The decomposed PV power signal is fed into the ANFIS model for outputting short-term PV power; different functions of wavelet mother were used (Haar, Daubechies, Coiflets, and Symlets). The achieved results demonstrate that the developed combination delivers high performance. Furthermore, they found that coif2-ANFIS and sym4-ANFIS offer low forecasting errors compared to all studied models.

Zhang et al.^46^ utilized a hybrid deep learning model coupled with WD and Artificial neural networks (ANN) to improve solar power plant output forecasts. They used WD as the network's transfer function and treats the model-weight, scaling-factor, and translation-factor as genetic individuals. The network's parameters are then obtained through independent optimization using a genetic algorithm and imported into the network. The authors found that the GA-WNN network performance outperforms conventional ANN models.

A hybrid deep LSTM network integrating wavelet packet decomposition (WPD) is proposed by Pengtao et al.^47^. Authors have utilized hybrid deep learning for 1-h-ahead PV power forecasting. The WPD algorithm is used to subdivide the initial PV power series. Four LSTM networks model are created for these sub-series. The performance of the WPD–LSTM method was demonstrated with a case study using data collected from an actual PV system in DKASC, Alice Springs, Australia. compared to individual LSTM, RNN, GRU, and MLP models, the WPD–LSTM model performs better in terms of predicting stability and accuracy.

### Variational mode decomposition

Zang et al.^48^, developed a new hybridization method based on variational mode decomposition (VMD) integrated with the DCNN model for PV power forecasting on a daily and hourly scale. Each component of the VMD technique, which originated from PV power, is transformed into a 2D image for further training in parallel channels of the DCNN model. The proposed forecasting methodology 2D-VMD-DCC proves its ability in forecasting PV power with high precision compared to SVM, GPR, MLP, VMD-SVM, SMD-GPR, and 1D-VMD-CNN. Netsanet et al.^49^ proposed a multi-stage forecasting model for daily PV power forecasting in China. The proposed methodology involves five main stages, firstly the time series of PV power is decomposed through the VMD algorithm into different components, then mutual information MI is employed to select the most pertinent input data. In the forecasting steps, the ANN model is employed with the ant colony optimization (ACO) technique for hyper-parameters tuning to output the desired PV power, another ANN model is utilized as a cascade forecasting using the previously estimated PV power as input for the final forecast. The forecasting ability of the proposed VMD-ACO-ANN was compared to the single ANN model and the combined ANN with the genetic algorithm GA-ANN model. The forecasting performance of the proposed technique in terms of statistical metrics outperforms the benchmarking models.

Wu et al.^50^, proposed a new hybridization methodology for very short-term PV power forecasting in Australia. In the first stage, the VMD technique is used to decompose the PV signal into different frequency bands, then the fast correlation-based filter (FCBF) algorithm is employed to select relevant input features, then a BILSTM model is used as the essence regressor. The proposed VMD-FCBF-BILSTM model proves its forecasting ability against conventional models.

A new decomposition-combined model is proposed by Xie et al.^51^, the proposed combination method is based on the use of the VMD algorithm to decompose the PV power data into different frequency components, then Deep Belief Network DBN model is used to forecast the high-frequency components and Autoregressive model is used to forecast low-frequency components. The achieved results prove that the developed technique gives excellent performance compared to the basic models.

Korkmaz et al.^52^, proposed a new forecasting model SolarNet for short PV power forecasting in Australia Solar Centre. The proposed SolarNet is dependent on the use of the VMD decomposition algorithm and CNN model. The input data are; solar radiation, active power data, temperature, and humidity. The proposed CNN model consists of two structures, max-pooling, and pooling blocks to boost the forecasting effectiveness. The achieved results show that the proposed model outperforms other deep learning techniques for 1-h ahead forecasting.

Zhang et al.^53^ developed an integrated model for PV power assessment through the hybridization of the VMD algorithm, CNN, and BiGRU. The VMD algorithm decomposed the PV power series into several sub-modes. An input feature matrix is built using the antecedent values and various environmental parameters. The CNN technique is then used to model the fundamental input–output relation between the feature matrix and the target value. Next, the BiGRU network is used to forecast the next PV power. Combining the forecasting values of the sub-modes yields the exact forecasted PV power result. The results showed that the VMD-CNN-BiGRU model outperformed the EEMD-CNN-BiGRU model.

Korkmaz et al.^54^ proposed the use of SolarNet which is a one-dimension convolutional neural network model for short-term PV output power forecasting over various weather conditions. The experiments are carried out as a case study utilizing a 23.40 kW PV dataset from the Desert of Australia. The measured meteorological data are used as input parameters to the SolarNet model. To create deep input feature maps, the power data is split into sub-components using the VMD approach, followed by a data preparation and reconstruction procedure. The achieved results show that the VMD-CNN forecasting technique delivers excellent performance.

## Contributions of the paper

Based on the analysis of relevant literature, we may infer that decomposition ensemble-learning techniques for PV power forecasting provide promising outcomes for further research. Firstly, the majority of developed models employ only one decomposition technique as a pre-processing step in the forecasting process of PV power. Secondly, instead of utilizing the decomposed PV power time series directly, the use of feature selection is a necessary step in improving prediction performance. Decomposition models are a feasible way of enhancing forecasting precision, based on the advantages and limits indicated in the current research. This research makes use of the concept of hybrid forecasting and introduces a new way of predicting PV power to fill the gap in the existing literature on this problem. The proposed hybridization mechanism is based mainly on the following combination: ensemble decomposition methods, multi-scale feature fusing, feature selection approach, and deep learning model.

The following section outlines the work's main novelty:

- A new fusion strategy IF-VMD-FSRA-CNN for short PV power assessment is optimized to generalize and improve predicting efficiency in a variety of meteorological conditions.
- The assessment and interpretation of several decomposition methods for short-term PV-plants power forecasting.
- The fusing of multi-scale features from the employed decomposition techniques for boosting the forecasting precision of PV power output.
- Using a feature selection technique allows users to choose the most meaningful intrinsic function IMFs from various multi-scale methodologies, which improves forecasting ability and minimizes the dimensionality of the data.
- The deep CNN model is capable of deriving deeper characteristics from the fused multi-scale information to provide the appropriate PV output power.
- Several tests were conducted, and the findings show that the suggested fusion mechanism produces more precise forecast outcomes than its counterparts models.

In comparison to previous research, the suggested forecasting methodology's efficiency is confirmed and validated on three grid-connected PV power systems in Algeria with various climate conditions. The suggested solution that utilizes hybrid approaches has yielded the lowest forecasting error.

## Material and monitoring

### Summary of data

**Figure 1:**
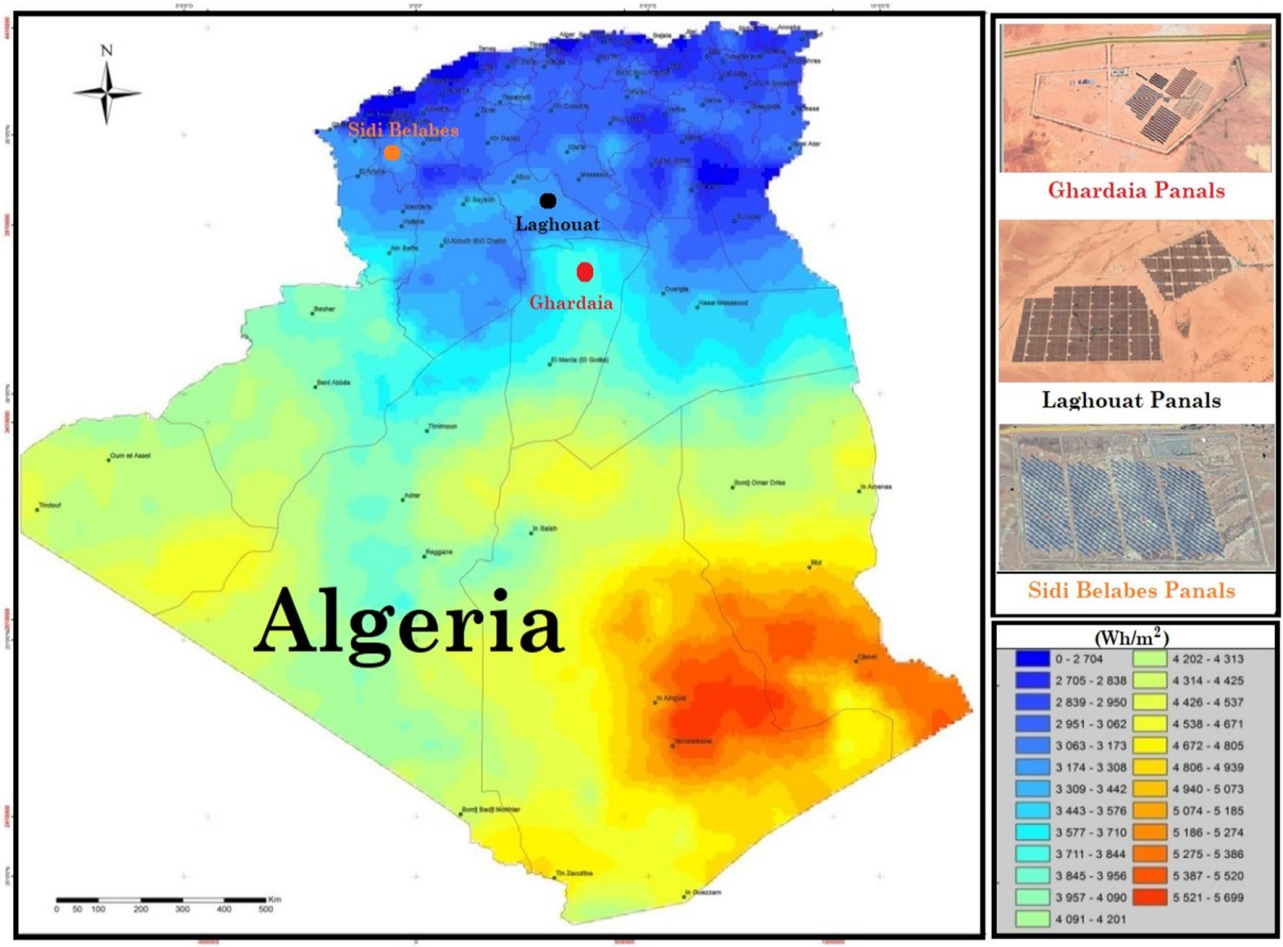
The geographical distribution of the three grid-connected PV-plants [Software-QGIS (A Free and Open Source Geographic Information System), Version 3.36.0 RC, Link: https://qgis.org/en/site/)] [Global Solar Atlas (Link: https://globalsolaratlas.info/map)].

Data from three photovoltaic power installed in three distinct locations covering different climatic conditions in Algeria were used in the study. The map in Fig. 1 depicts the geographic coverage of solar PV power plants employed in this research. The study area covers the three selected sites. The first PV plant station is Lekhneg, in the Laghouat region (33° 43′ 26.74″ N, 2° 48′ 45.27″ E)with a capacity of 60 MWp located in the south of Algeria^55^. The second grid-connected PV power is Oud Nechou in the Ghardaia region^56,57^, (32° 36′ 1.46″ N, 3° 42′ 3.42″ E) located also in the south of Algeria with a capacity of 1.1MWp. The third PV power station is Dhaya of Sidi Bel-Abbes (34° 41′ 32.23″ N, 0° 36′ 2.89″ O) located in the west of Algeria with a capacity of 12 MWp^58^.

**Figure 2:**
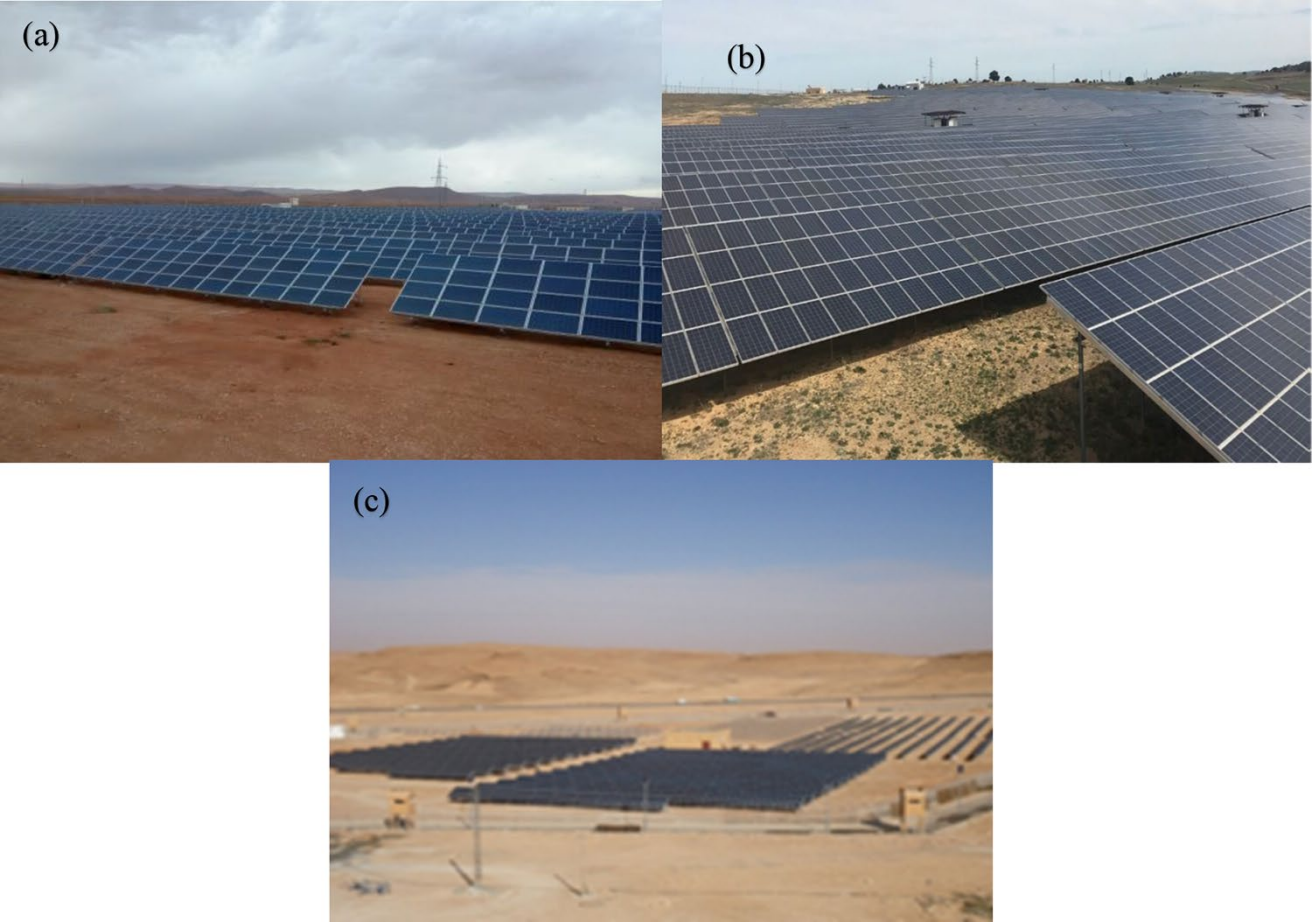
(**a**) Photo of the Laghouat site, (**b**) Photo of Sidi Bel Abbés site, (**c**) Photo of Ghardaia site.

The photovoltaic solar power plants of Lahgouat, Ghardaa, and Sidi Bel-Abbes were commissioned in (April 2015/2016), as part of the national renewable energy program, and are one of 21 identical stations installed across the country to produce 400 MW. The information regarding the type of photovoltaic modules, the area of photovoltaic plants, and the data period of measured PV power in each PV station are given in Table 1. The photographs of the studied PV plants can be viewed in Fig. 2.

**Table 1:** Information on the three studied PV plants under consideration.

PV solar fields	Data period	Area	Type of photovoltaic modules installed,
Laghouat I et II	2019–2020	60 Ha	Crystalline poly, Type: YL250P-29b
Sidi Bel Abbés	2019–2020	36.6 Ha	Crystalline poly, Deux Type: HSL60P6-PB-1-250
Ghardaïa piloté	2016–2017	10 Ha	Thin film (Cd-Te), Type : FIRST SOLAR FS-380
			Amorphous silicon (a-Si), Type : SCHOTT ASI 103
			Polycrystalline silicon, Type ATERSAA-235P
			Monocrystalline silicon, Type AtersaA-250 M

## Methodology

### Complete ensemble empirical mode decomposition with adaptive noise (CEEMD)

CEEMDAN is an extension of EMD. Modal aliasing could be further reduced by using adaptive white noise to increase decomposition effectiveness. It is possible to utilize CEEMDAN to analyze and handle non-stationary signals in time and frequency^59^. It can generate signal wave patterns of various sizes and create a series of data sequences with local features at various periods, where each component is a stationary IMF. Two criteria must be satisfied by each IMFs component: (1) the mean value of the envelope formed by its local maximum and minimum must be zero at any moment, and (2) the number of extreme points in the entire dataset must be the same as zero-crossing points or vary by at most one.

For a time-series sequence $\left(t\right)$ , the key steps of CEEMDAN decomposition are as follows:

- At time m, we introduce white noise ${W}^{m}\left(t\right)$ to the original signal ${X}^{m}\left(t\right)$.
  1 $${X}^{m}\left(t\right)=X\left(t\right)+{\varepsilon }_{0}{W}^{m}\left(t\right),m=\mathrm{1,2},\dots ,M$$
  where M represents the number of realizations.
- The first EMF $\overline{{IMF }_{1}} \left(t\right)$ is calculated by taking the average of the EMD:
  2 $$\overline{{IMF }_{1}} \left(t\right)=\frac{1}{M}\sum_{i=1}^{M}{IMF}^{1}\left(t\right)$$
  Then, the residual components are computed as:
  3 $${{\text{r}}}_{1}\left(t\right)=X\left(t\right)-\overline{{IMF }_{1}} \left(t\right)$$
- The signal ${r}_{1}\left(t\right)+{\varepsilon }_{1}{EMD}_{1}\left({W}^{m}\left(m\right)\right)$ is decomposed in its turn by the EMD algorithm to produce the second IMF and the corresponding residual as below:
  4 $$\overline{{IMF }_{2}}=\frac{1}{M}\sum_{i=1}^{M}{EMD}_{1}\left({r}_{1}\left(t\right)+{\varepsilon }_{1}{EMD}_{1}\left({W}^{i}\left(t\right)\right)\right)$$
  5 $${r}_{2}\left(t\right)={r}_{1}\left(t\right)-\overline{{IMF }_{2}}$$
- Repeat these steps to obtain the k-th residual and k + 1-th IMF component:
  6 $${r}_{k}\left(t\right)={r}_{k-1}\left(t\right)-\overline{{IMF }_{2}}\left(t\right)$$
  7 $$\overline{{IMF }_{k+1}}\left(t\right)=\frac{1}{M}\sum_{i=1}^{M}{EMD}_{1}\left({r}_{k}\left(t\right)+{\varepsilon }_{k}{EMD}_{k}\left({W}^{i}\right)\right)$$
  where ${EMD}_{k}$ (■) indicates the k-th IMF mode.
- Repeat Eqs. (6) and (7) until the remaining signal is unsuitable for being further decomposed through the EMD algorithm and then terminate the algorithm.

Finally, the time series X(t) decomposition result may be written as follows:

8 $$x\left(t\right)=\sum_{i=1}^{k}\overline{{IMF }_{k}}\left(t\right)+R\left(t\right)$$

### Variation mode decomposition (VMD)

The VMD technique is one of the most recent signal processing methods.. The VMD method is a non-recursive and adaptive decomposition process proposed for non-stationary signals. The VMD algorithm is similar to an adaptive Winer filter bank, which can split a given signal into a set of center frequencies with a restricted frequency range different from the EMD algorithm. The essence of the VMD technique is to decompose a given input signal into several sub-models that have a restricted bandwidth and a certain level of sparseness in bandwidth^60^.

Constructing and resolving variation issues is the primary focus of this approach. Solving constrained variation optimization issues is stated as:

9 $$ \begin{array}{*{20}l}    {min_{{\left\{ {uk} \right\},\left\{ {wk} \right\}}} \left\{ {\sum _{k} \left\| {\delta _{t} \left[ {\left( {\delta \left( t \right) + \frac{j}{{\pi t}}} \right)*u_{k} \left( t \right)} \right]e^{{ - jw_{k} t}} } \right\|_{2}^{2} } \right\}} \hfill  \\    {s.t.\sum\limits_{k} {u_{k} } \left( t \right) = f\left( t \right)} \hfill  \\   \end{array}  $$

Wher ${u}_{k}\left(t\right)$ represent the model function of the signal,$\left\{uk\right\}$ is the model group {$u1$ ,$u2$ ,…,$u3$ },$wk$ is the central frequency of the k-th mode of the signal,$\left\{uk\right\}$ indicate the center frequency of these decomposed modes,f(t) and $\delta \left(t\right)$ represent the input signal and unit pulse function respectively.

Utilizing quadratic penalty factor and Lagrangian multiplier Eq. (1) can be developed as:

10 $$L\left(\left\{{u}_{k}\right\},\left\{{w}_{k}\right\},\lambda \right)=\psi \sum_{k}{\left\Vert {\partial }_{t}\left[\left({\partial }_{t}+\frac{j}{\pi t}\right)*{u}_{k}\left(t\right)\right]{e}^{-j{w}_{k}t}\right\Vert }_{2}^{2}+{\Vert f\left(t\right)-\sum_{k}{u}_{k}\left(t\right)\Vert }_{2}^{2}+\langle \lambda \left(t\right),f\left(t\right)-\sum_{k}{u}_{k}\left(t\right)\rangle $$

The changing orientation multiplication technique (ADMM) is employed to solve Eq. (10). The ${u}_{k}$ can be described as:

11 $${\widehat{u}}_{k}^{n+1}\left(w\right)=\frac{\widehat{f}\left(w\right)-{\sum }_{i=1}^{k-1}{\widehat{u}}_{i}^{n+1}\left(w\right)-{\sum }_{i=k+1}^{k}{\widehat{u}}_{i}^{n}\left(w\right)+\frac{{\stackrel{`}{\lambda }}^{n}\left(w\right)}{2}}{1+2\psi {\left(w-{w}_{k}^{n}\right)}^{2}}$$

where n represents the iteration numbers, $\widehat{f}\left(w\right)$, ${\widehat{u}}_{k}^{n+1}\left(w\right)$, ${\widehat{u}}_{i}\left(w\right)$ and ${\stackrel{`}{\lambda }}\left(w\right)$ indicate the form after $f\left(wt\right)$, ${u}_{k}^{n+1}\left(t\right)$, ${u}_{k}\left(t\right)$, and $\lambda \left(t\right)$ Fourier transform^61^.

12 $$ w_{k}^{{n + 1}}  = \psi \sum\limits_{k} {\delta _{t} } \left\| {\left[ {\left( {\delta \left( t \right) + \frac{j}{{\pi t}}} \right)*u_{k} \left( t \right)} \right]e^{{ - jw_{k} t}} } \right\|_{2}^{2}  $$

### Wavelet packet decomposition (WPD)

The WPD technique is traditional signal processing, which split a given signal into its adequate and detailed elements. Wavelet basis and decomposition levels have a significant impact on the WPD's performance. There are two kinds of WPD: the wavelet transform in its continuous and discrete forms. The following is a description of the continuous wavelet transform^62^:

13 $${CWT}_{f}\left(a,b\right)=\langle f\left(t\right),{\psi }_{a,b}\left(t\right)\rangle =\int\limits_{-\infty }^{+\infty }f\left(t\right){\psi }^{*}\left(\frac{\left(t-b\right)}{a}\right)/\sqrt{a}dt$$

where f(t) and ${\psi }\left(t\right)$ represent the input signal and the mother wavelet, respectively. The term b indicates the translation factor and a represents the scale factor and * denotes the conjugate complex factor. The discrete form of a and b in DWT can be summarized as follows:

14 $$\left\{\begin{array}{c}{a=2}^{j}\\ {b=k2}^{j}\end{array}\right.$$

### Iterative filtering decomposition method (IF)

Recently, the iterative filtering technique is proposed to be an alternative to the EMD method and its variants^2^ for signal processing with adaptive filtering to enforce the convergence of IF. The primary variance between the IF approach and EMD and its alternative is that the moving average of a specific signal $f\left(x\right),x\in {\mathbb{R}}$ is accomplished by employing the convolution of $f\left(x\right)$ with a specific law pass filter^63^. Let's consider a signal $\left(x\right),x\in {\mathbb{R}}$ and $\mathcal{L}\left(f\right)$ represent the moving average of the input signal which can be estimated by^64^:

15 $$\mathcal{L}\left(f\right)\left(x\right)=\int\limits_{-l}^{l}f\left(x+t\right)a\left(t\right)dt$$

The term $a\left(t\right)$ indicate the double average filter given by:

16 $$a\left(t\right)=\frac{l+1-\left|t\right|}{{\left(l+1\right)}^{2}} ,t\in \left[-\mathrm{1,1}\right]$$

In IF methodology the operator $\mathcal{L}\left(f\right)$ is obtained by the convolution of the signal $f\left(x\right)$ by some specific filter $W$.

Let’s define our operator as: ${S}_{1,n}\left({f}_{n}\right)$=${f}_{n}-{\mathcal{L}}_{1,n}\left({f}_{n}\right)={f}_{n+1}$ which shows the alteration part of ${f}_{n}$, the first IMF_1_ can be obtained by Eq. (17):

17 $${IMF}_{1}=\underset{n\to \infty }{{\text{lim}}}{S}_{1,n}\left({f}_{n}\right)$$

Apply the same method to the rest $f-{IMF}_{1}$ until it becomes a trend signal, which implies it has just one local maximum or minimum. All IMF components are driven by two loops in the proposed IF methodology: an inner and an outer loop.

### Time-varying filter-based empirical mode decomposition algorithm (TVF-EMD)

Time-varying filter-based empirical mode decomposition (TVF-EMD) is a newly established variant of the classic (EMD) suggested by Li et al.^65^. By adopting a B-spline approach filter to cope with shifting processes, the suggested TVF-EMD demonstrates its efficacy in resolving the shortcomings of the standard EMD algorithm. The fundamental concept of the suggested TVF-EMD relies on the fixed cutoff frequency and then follows with a time-varying filtering technique. The following are the key phases of the proposed TVF-EMD^65,66^:

- 1: Estimate the maximum point and identify it for a given signal $x\left(t\right)$ as :
- 2: Determine all instances of intermittency that meet the following criteria:

$\frac{max\left({\varphi }_{bis}{\prime}\left({u}_{i}:{u}_{i+1}\right)\right)-min({\varphi }_{bis}{\prime} ({u}_{i}:{u}_{i+1} ))}{min\left({\varphi }_{bis}{\prime}({u}_{i}:{u}_{i+1})\right)}>\rho$, where the position of ${u}_{i}$ can be considered as an intermittence :${e}_{j}\left(j=\mathrm{1,2},3,\dots \right),{e}_{j}={u}_{i}$.

- 3: For each state of ${e}_{j}$ , there are two alternative positions placed on the falling edge or rising of ${\varphi }_{bis}{\prime}\left(t\right)$, if ${\varphi }_{bis}{\prime}\left({u}_{i+1}\right)>{\varphi }_{bis}{\prime}\left({u}_{i}\right)$,${\varphi }_{bis}{\prime}\left({u}_{i}\right)$ can be viewed as a floor. Although, if ${\varphi }_{bis}{\prime}\left({u}_{i+1}\right)<{\varphi }_{bis}{\prime}\left({u}_{i}\right)$, then ${\varphi }_{bis}{\prime}\left({u}_{i}\right)$ can be found on its falling edge.
- 4: Interpolatebetween the maxima to perform the adaptation of the local cut-off frequency.

where $\left({\varphi }_{bis}{\prime}\left(t\right)\right)$ specifies the cutoff frequency; a more detailed explanation may be found in^65^. Furthermore, the ending condition mentioned in the sifting step is as follows:

18 $$\theta \left(t\right)=\frac{{B}_{Loughlin}\left(t\right)}{{\varphi }_{avg}\left(t\right)}$$

where ${B}_{Loughlin}\left(t\right)$ represent Loughlin instantaneous bandwidth and ${\varphi }_{avg}\left(t\right)$ displays the weighted average of the different components' instantaneous frequencies^65,66^. Actually, with the stated bandwidth cutoff $\varepsilon$, the term "signal local narrow-band" is described as:$\theta \left(t\right)\le \varepsilon$ , in which TVF-EMD spliting performance will be adequately adjusted.

### Convolution neural network (CNN)

Deep learning techniques as a subfield of machine learning (ML) have received considerable attention from the scientific community in recent years. The CNN model, which is a significant deep-learning architecture inspired by the natural visual system of mammals is developed by LeCun^67–69^proposed. Deep CNNs can be built using the backpropagation technique to perform tasks such as classifying handwritten digits. Deep CNNs have dominated various computer vision and image processing applications, and recent applications of CNNs for time series prediction have generated highly promising results^70,71^. Convolution, pooling layer, and fully connected layers are the main building blocks that CNN's use to perform spatial hierarchies of features. The deep CNN model is mostly well adopted with time series signals for various regression and classification problems as introduced in^72^. The CNN models perform the optimization performance with less memory consumption, there is already a connection between the neurons in the adjacent layer of the fully connected networks. The hyper-parameters of Deep CNN model can be reduced efficiently; also, CNN models are able to extract special features with different lengths. Figure 5 displays an illustration of a CNN model used for time series forecasting employing univariate time-series data as input, we note that CNN's are more suitable suited for multivariate time series with features extracted through convolution and pooling layers.

### Forward stepwise regression algorithm (FSRA)

Forward Stepwise Regression Algorithm (FSRA) is a systematic method used in statistical modeling to select the most relevant variables for a predictive model. This algorithm falls under the category of stepwise regression, which includes both forward selection and backward elimination methods. FSRA specifically uses the forward selection approach, where the model building process starts with no variables and then adds them one at a time based on specific criteria. The main steps of FSRA are as follow:

1. *Initialization* Begin with a model containing no predictors. This means starting with a simple model that only includes the intercept .
2. *Variable Selection* At each step, consider adding each variable that is not already in the model. The choice of which variable to add is based on a predetermined criterion, usually the one that results in the most significant improvement to the model. Common criteria include the lowest p-value in a t-test for the coefficient, the largest increase in R^2^, or a significant decrease in the Akaike Information Criterion (AIC) or Bayesian Information Criterion (BIC).
3. *Model Evaluation* After adding the new variable, evaluate the model to ensure it significantly improves the model's performance. This can involve checking for statistical significance, improvements in goodness-of-fit measures, or cross-validation performance.
4. *Iteration* Repeat the variable selection and model evaluation steps, adding one variable at a time, until no further significant improvements can be made to the model.
5. *Final Model* The result is a model that includes a subset of the available variables that best explain the variation in the response variable, based on the criteria used for variable selection.

### The hybrid-forecasting model

**Figure 3:**
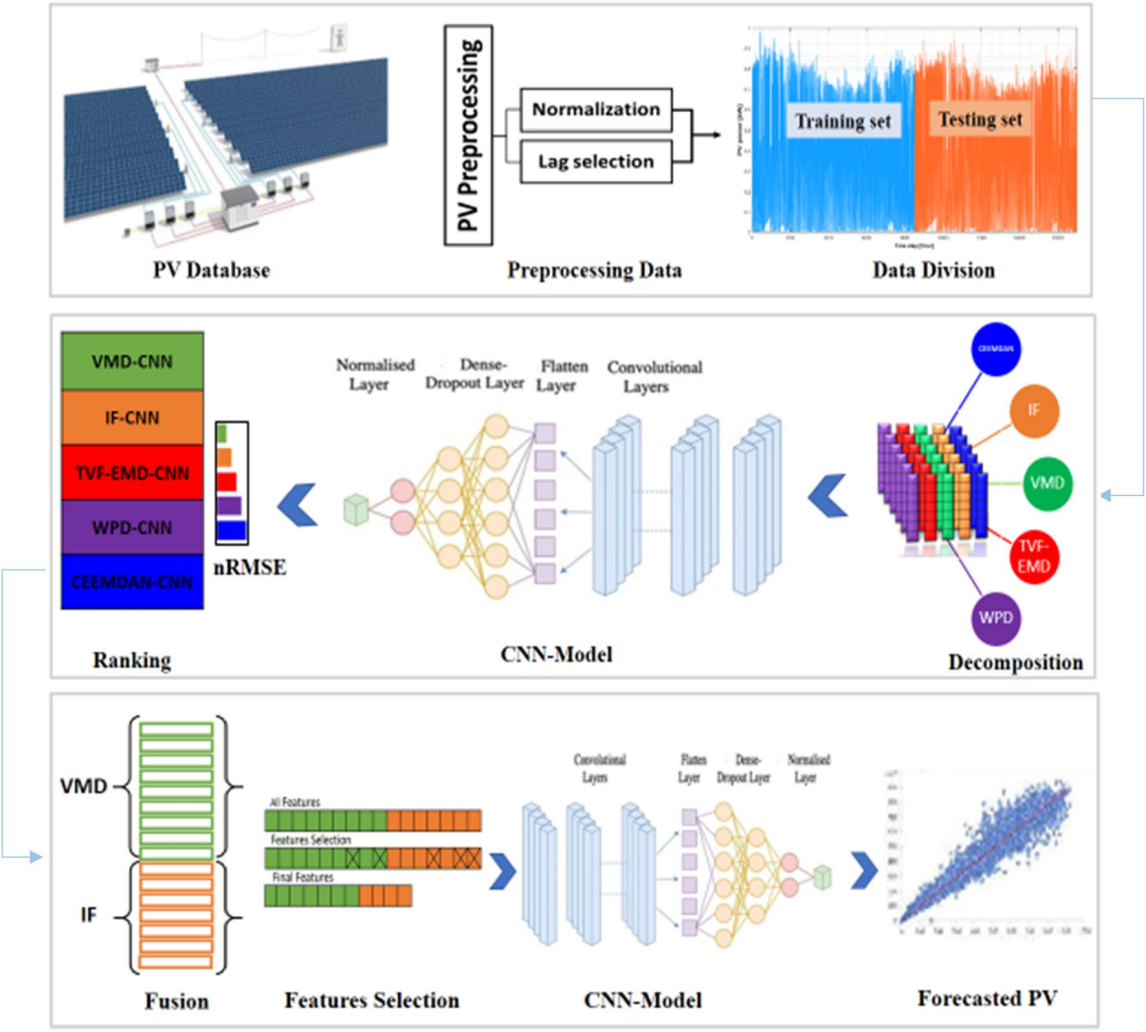
Flowchart of the proposed fusing strategy.

The main configuration of the suggested fusion strategy is presented in Fig. 3. Furthermore, the following are the major phases involved in the construction of the combined IF-VMD-FSRA-CNN forecasting model:

- *Step 1* Collect and analyze PV power data to generate training and testing sets. The training sets are utilized for model tuning (Hyper-parameter setting), while the testing sets are used for model assessment.
- *Step 2* Using the decomposition techniques, divide the training and testing sets into a varied number of IMFs elements. The nonlinearity and non-stationarity characteristic of the actual data might be efficiently used addressed using these decomposition methodologies.
- *Step 3* Individual use of each decomposition method for PV power forecasting and then fusing them to increase the forecasting accuracy.
- *Step 4* A feature selection technique is used to identify and rank the most important IMFs components for PV power forecasting.
- *Step 5* The identified IMFs sequence is deployed to train the deep CNN forecasting model as input variables.
- *Step 6* After that, the fully trained CNN model is utilized to evaluate forecasting efficiency on the test dataset.
- *Step 7* Examine the findings and assess the effectiveness of the three grid-connected under consideration.

It is feasible to demonstrate that the suggested integrated approach is intended to deal with a variety of qualities found that occur in the real world. Particularly, decomposition strategies were used to cope with the data's non-stationarity. The noisy and unnecessary IMFs components are then removed using a feature selection approach. The non-linear CNN model is then used to model the PV power output.

## Performance metrics

Five quality evaluations were chosen to quantify the impact of the suggested forecasting approach^8,69,73–75^ expressed in Table 2 as:

**Table 2:** Statistical metrics for forecasting.

Error metric	Equation
RMSE	$RMSE=\sqrt{\frac{1}{n}\sum_{i=1}^{n} {\left(\overline{H }-H\right)}^{2}}\quad\quad(18)$
nRMSE	$RMSE=\frac{RMSE}{\sum_{i=1}^{n}H}\quad\quad(19)$
MABE	$MABE=\frac{1}{n}\sum_{i=1}^{n}\left|\overline{H }-H\right|\quad\quad(20)$
nMAE	$NMAE=\frac{\sum_{i=1}^{n}\left|\overline{H }-H\right|}{\sum_{i=1}^{n}H}\quad\quad(21)$
r	$r=\frac{\sum_{i=1}^{n}\left(\overline{H }-mean\left(\overline{H }\right)\right)\left(H-mean\left(H\right)\right)}{\sum_{i=1}^{n}{\left(\overline{H }-mean\left(\overline{H }\right)\right)}^{2}{\left(H-mean\left(H\right)\right)}^{2}}\quad\quad(22)$

## Results and discussion

Precise short-term assessments of PV power are crucial for ensuring the production and stability of necessary power system capacity. This section intends to present the simulation results for the suggested technique using multiple techniques. These strategies are further divided into three phases. The primary concept is to create a combination of different decomposition strategies with a deep learning approach for PV power forecasting, and then apply the suggested strategy to diverse time series forecasting. We start by assessing the potential of extracting multi-frequency features with the suggested decomposition techniques and evaluate each decomposition method separately on three grid-connected photovoltaic stations. The suggested combination methodologies have been adjusted for a horizon of 15 min, and a half hours ahead of PV power output. The suggested combination technique is assessed and verified on three separate PV power data sets, with 50% of each analyzed data set employed for training and the remainder for assessing forecasting ability. Solar irradiation, temperature, and the angle at which PV arrays are installed all influence PV power production. The association between previous PV power and future output PV power was the only focus of the present research. The concept of trial and error was used to evaluate the effect of different delays and determine the acceptable number of delays.

The PV power and its previously decomposed elements with optimum lag identification are the target outputs for training the supervised decomposition-deep learning method in this work. For each analyzed region, data pre-processing is the same. To validate the integrated model's forecasting ability, the hybridization approaches were compared to stand-alone models (GPR, LS-SVM, ELM). All models were trained and tested using the same methodology. The quantitative findings of the assessment criteria for each decomposition strategy and their counterpart models are depicted in Tables 3, 4, and 5 for Laghouat, Sidi Bel Abbés, and Ghardaia stations, respectively. Bold numbers reflect the most accurate estimation. during the forecasting process. According to the findings reported, in Tables 3, 4, and 5, various observations may be inferred. Starting with the results delivered by hybrid approaches, where we can see that these latter significantly exceed the basic models in terms of statistical metrics^75^. Moreover, the hybrid approaches generate PV power output with high precision. It observed that the nRMSE error generated by all decomposition techniques is less than 10% in all analyzed areas whereas the nRMSE of individual models ranged from 22 to 39%. It can be also observed that all decomposition algorithms provide different forecasting errors which vary within the range of nRMSE = [0.61–7.44] in all studied stations. That is, the proposed combination can generate a 30-min and 15-min ahead PV power output with acceptable accuracy better than stand-alone models. The proposed VMD-CNN and IF-CNN models, followed by TVF-CNN, WPD-CNN, and CEEMDAN-CNN models achieve the best forecasting precision in all studied regions. The VMD-CNN and IF-CNN models provide the highest average PV power production, which will be evaluated as the best candidate algorithm for multiscale fusing. For a more comprehensive view of the model's precision, a statistical representation is also required in order to have a better comprehension of model precision.

**Table 3:** Performance of different decomposition techniques for PV power, Laghouat region.

Forecasting Horizon		Model	RMSE(W)	nRMSE	MABE	nMAE	r (%)
30-min	Stand-alone models	GPR-model	6533	26.73	3560.4	14.86	0.914
		LS-SVM-model	5530	22.63	3175	12.953	0.937
		ELM-model	5269	21.56	3035	12.38	0.94
	Hybrid-models	WPD-CNN	917.61	3.755	614.65	2.512	0.998
		CEEMDAN-CNN-model	1791.7	7.3332	1179.9	4.8132	0.993
		IF-CNN-model	268.82	1.1002	172.23	0.7046	0.999
		TVF-EMD-CNN-model	685.39	2.80	429.26	1.764	0.999
		VMD-CNN-model	**151.16**	**0.6185**	**112.75**	**0.4611**	**0.999**

Significant values are in bold.

**Table 4:** Performance of different decomposition techniques for PV power, Sidi Bel Abbés region.

Forecasting Horizon		Model	RMSE(KW )	nRMSE	MABE	nMAE	r (%)
15-min	Stand-alone models	GPR-model	1372.427	30.774	828.29	17.76	0.885
		LS-SVM-model	1343.370	30.122	818.24	17.21	0.894
		ELM-model	1176.392	26.378	771.95	16.40	0.923
	Hybrid-models	WPD-CNN	178.083	3.993	127.01	2.851	0.998
		CEEMDAN-CNN-model	294.281	6.598	213.23	4.786	0.994
		IF-CNN-model	39.645	0.889	27.470	0.615	0.999
		TVF-EMD-CNN-model	140.883	3.159	106.47	2.397	0.998
		VMD-CNN-model	**35.984**	**0.806**	**27.227**	**0.610**	**0.999**

Significant values are in bold.

**Table 5:** Performance of different decomposition techniques for PV power, Ghardaia region.

Forecasting horizong		Model	RMSE(KW )	nRMSE	MABE	nMAE	r (%)
30-min	Stand-alone models	GPR-model	201.738	39.647	124.70	29.891	0.782
		LS-SVM-model	122.785	24.131	91.744	20.043	0.924
		ELM-model	113.626	22.331	80.818	17.234	0.926
	Hybrid-models	WPD-CNN	24.314	4.778	17.388	3.43	0.996
		CEEMDAN-CNN-model	37.858	7.440	23.539	4.64	0.990
		IF-CNN-model	**10.319**	**2.028**	**7.441**	**1.469**	**0.999**
		TVF-EMD-CNN-model	20.694	4.067	15.497	3.092	0.998
		VMD-CNN-model	11.455	2.251	8.433	1.674	0.999

Significant values are in bold.

**Figure 4:**
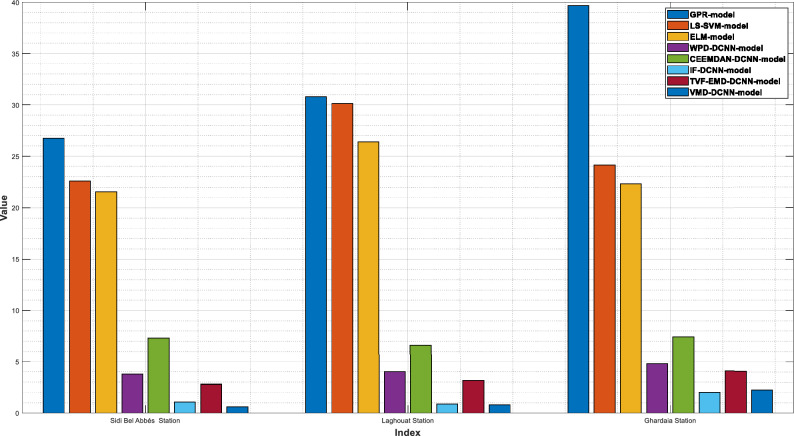
Forecasting results overall studied regions in terms of nRMSE.

As shown in Fig. 4, the VMD-CNN model ranked as the best forecasting model for Laghouat and Sidi bel abbes PV station followed by the IF-CNN model. In the case of Ghardaia PV station, we observe that the IF-CNN model provides the best forecasting performance followed by the VMD-CNN model. All hybrid models, which have satisfactory convergence, perform better than individual models. On the contrary, the results achieved by single models (LS-SVM, GPR, and ELM-model) illustrate that these models are ineffective as compared to hybrid models.

**Figure 5:**
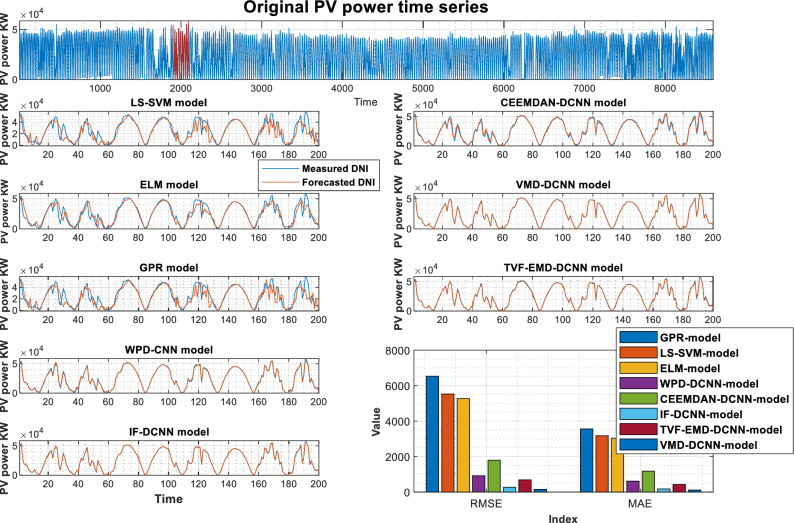
Forecasting results of various models Laghouat PV stations.

**Figure 6:**
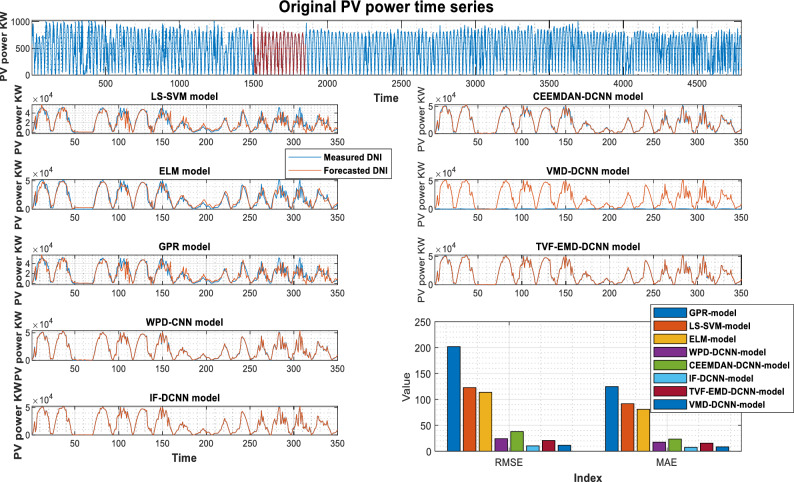
Forecasting results of various models Sidi Bel Abbés PV stations.

**Figure 7:**
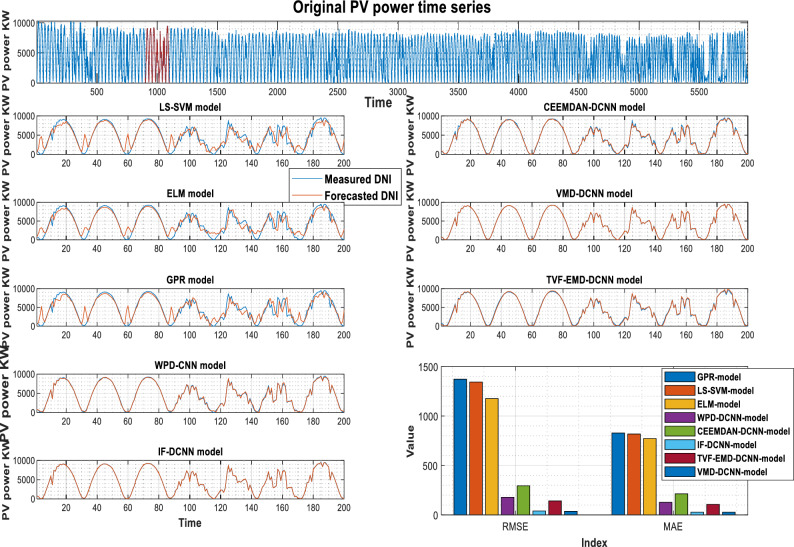
Forecasting results of various models (Ghardaia PV stations).

Figures 5, 6, and 7 show the actual and estimated PV power output on sunny and cloudy days patterns for the three studied regions. One can observe from the comparison of PV power forecasting that the forecasted PV power curve of stand-alone models (GPR, ELM, and LS-SVM model) show a remarkable gap between true and modeled values of PV power under different weather conditions. In both specific cases (sunny, and cloudy days), the results produced using hybrid decomposition ensemble learning techniques outperform those obtained with stand-alone models. Another relevant point is that the forecasting trend of the five developed hybrid models is identical to the actual data. For the three-studied PV station, the IF-CNN model and VMD-CNN model generate the closest PV power estimate to the real PV power data. In addition, the sub-figures show that the proposed VMD-CNN model and IF-CNN model can accurately match the trend and features of real PV power than the benchmarking models. The same figures may be used to infer additional information.

The outcomes are analyzed by considering all information investigated with all forecasting ranges. Because of the large number of numerical results in Tables 3, 4 and 5, it is necessary to investigate several elements of the forecasting model efficiency. On the resulting nRMSE metric, the Friedman and Nemenyi hypothesis testing^76^ was employed. Friedman test is a statistical method used to rank various models in classification or regression tasks. This test is particularly useful when these models exhibit inconsistent performance, i.e., their effectiveness varies from one dataset to another, making it challenging to determine the best model for a specific application. The Friedman-Nemenyi test is an effective statistical approach that aids in the ranking and comparison of the models used.

**Figure 8:**
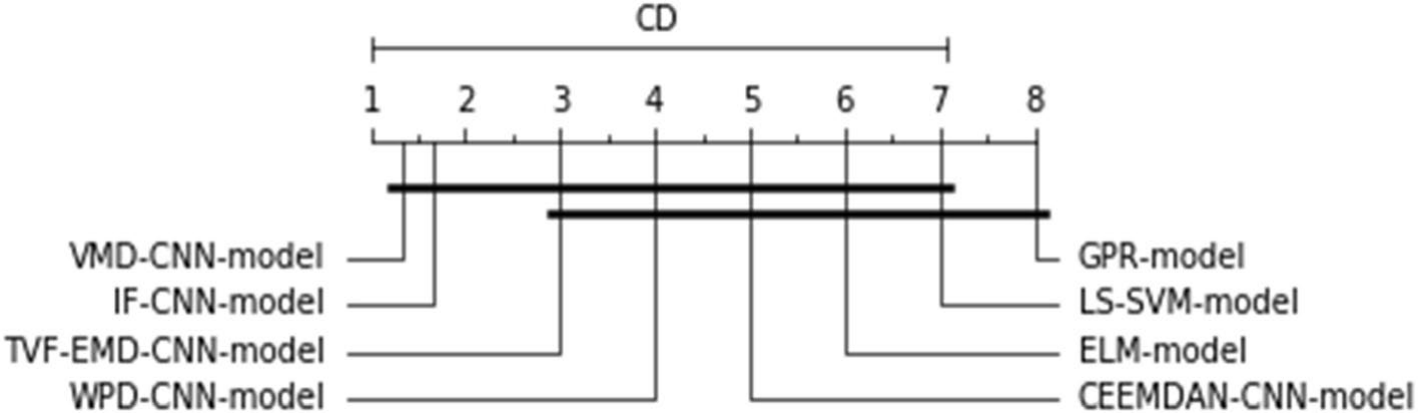
The Friedman-Nemenyi test applied to all datasets and methodologies.

Friedman's test has been employed to score the proposed models throughout all forecasting horizons and analyzed PV plants. If the order difference is statically important, then a Nemenyi hypothesis test is conducted by comparing all forecasting models pair-by-pair. The RMSE, as a dependent scale statistic, cannot be used for model comparison across several datasets^77^. The previous study on time series forecasting has reported a similar concept, which can arise when alternative error metrics are utilized^78^. As a result, the test is conducted totally with the nRMSE metric, and the results are shown in Fig. 8.

As can be shown, after employing the Friedman-Nemenyi test statistic with 95% certainty, the suggested hybridization approach VMD-CNN-model delivers the best overall forecasting performance, outperforming hybrid and classic models when all-time series data is used.

The second part of our experiment investigates the fusing of multi-scale features two by two to improve the effectiveness of the forecasting performance of PV power output. Tables 7, 8, and 9, show the achieved results of the suggested strategy. The forecasting combination’s performance was examined using distinct day types. The results of different fusing combinations are shown in Tables 6, 7, and 8, the best outcomes are shown in bold. According to the numerical indicators, all proposed combination methods succeed in increasing the forecasting accuracy compared to the conventional forecasting model and individual decomposition techniques combined with the deep-CNN model, resulting in a reduced nRMSE = [0.44%-4] for all studied regions.

**Table 6:** Performance of fusing different decomposition techniques for PV power, Laghouat.

Forecasting horizon lg	Model	RMSE	nRMSE	MABE	nMAE	r (%)
30-min	TVF-EMD-WPD-CNN	445.491	1.822	305.80	1.250	0.9996
	TVF-EMD-CEEMDAN-CNN	723.993	2.962	522.682	2.145	0.9989
	TVF-EMD-IF-CNN	307.289	1.257	209.27	0.856	0.9998
	CEEMDAN-WPD-CNN	837.946	3.428	597.398	2.453	0.9986
	IF-WPD-CNN	350.879	1.435	233.637	0.955	0.9997
	IF-CEEMDAN-CNN	426.60	1.745	302.356	1.233	0.9996
	VMD-TVF-EMD-CNN	246.41	1.008	180.958	0.740	0.9998
	VMD-CEEMDAN-CNN	200.044	0.818	151.228	0.619	0.9999
	VMD-WPD-CNN	192.753	0.788	142.129	0.581	0.9999
	VMD-IF-CNN	**108.07**	**0.442**	**73.717**	**0.3015**	**0.9999**

Significant values are in bold.

**Table 7:** Performance of fusing different decomposition techniques, Sidi Bel Abbés.

Forecasting horizon sd	Model	RMSE	nRMSE	MABE	nMAE	r (%)
15-min	TVF-EMD-WPD-CNN	113.029	2.534	86.533	1.944	0.999
	TVF-EMD-CEEMDAN-CNN	120.668	2.706	94.704	2.119	0.999
	TVF-EMD-IF-CNN	75.739	1.698	57.543	1.294	0.999
	CEEMDAN-WPD-CNN	160.444	3.597	120.773	2.707	0.998
	IF-WPD-CNN	61.605	1.381	44.196	0.991	0.999
	IF-CEEMDAN-CNN	72.700	1.630	54.39	1.222	0.9996
	VMD-TVF-EMD-CNN	98.461	2.207	72.880	1.638	0.999
	VMD-CEEMDAN-CNN	43.647	0.978	33.204	0.745	0.999
	VMD-WPD-CNN	38.833	0.870	29.370	0.658	0.999
	VMD-IF-CNN	**21.288**	**0.477**	**16.359**	**0.366**	**0.999**

Significant values are in bold.

**Table 8:** Performance of fusing different decomposition techniques, Ghardaia.

Forecasting horizon sd	Model	RMSE	nRMSE	MABE	nMAE	r (%)
30-min	TVF-EMD-WPD-CNN	19.136	3.760	13.619	2.704	0.99
	TVF-EMD-CEEMDAN-CNN	17.810	3.500	13.474	2.683	0.9987
	TVF-EMD-IF-CNN	11.742	2.307	9.0245	1.785	0.9995
	CEEMDAN-WPD-CNN	20.356	4.000	14.225	2.795	0.9974
	IF-WPD-CNN	12.252	2.408	8.749	1.729	0.999
	IF-CEEMDAN-CNN	11.426	2.245	8.093	1.594	0.999
	VMD-TVF-EMD-CNN	13.263	2.606	9.875	1.968	0.9995
	VMD-CEEMDAN-CNN	7.939	1.560	6.015	1.187	0.999
	VMD-WPD-CNN	10.331	2.030	7.792	1.542	0.999
	VMD-IF-CNN	**7.8707**	**1.546**	**5.734**	**1.133**	**0.999**

Significant values are in bold.

Another important remark is that combining decomposition approaches results in a significant improvement compared with the case of using each decomposition technique individually. As expected the integration of IF and VMD algorithm with deep-CNN-model provides high forecasting precision for all grid-connected PV stations. These outcomes show clearly that combining multi-scale features is highly beneficial for improving the forecasting precision of PV power^87,88^.

Another important observation is that VMD-CEEMDAN-CNN, VMD-WPD-CNN, and VMD-IF-CNN models are ranked as the best forecasting models for PV power output production, with a significant improvement achieved by the VMD-IF-CNN model for all studied PV power stations.

**Figure 9:**
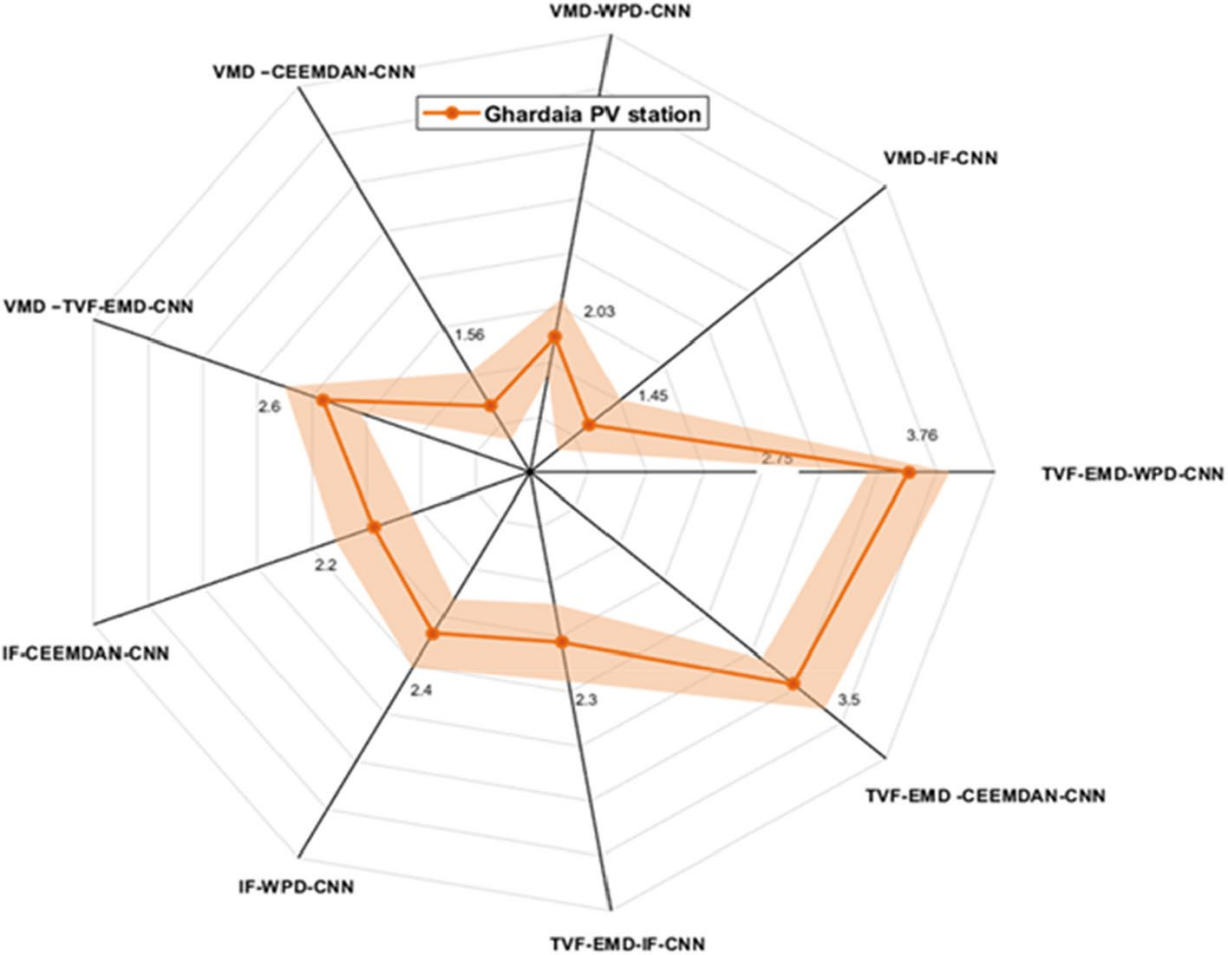
Performance comparison of different decomposition algorithms in terms of nRMSE (Ghardaia).

**Figure 10:**
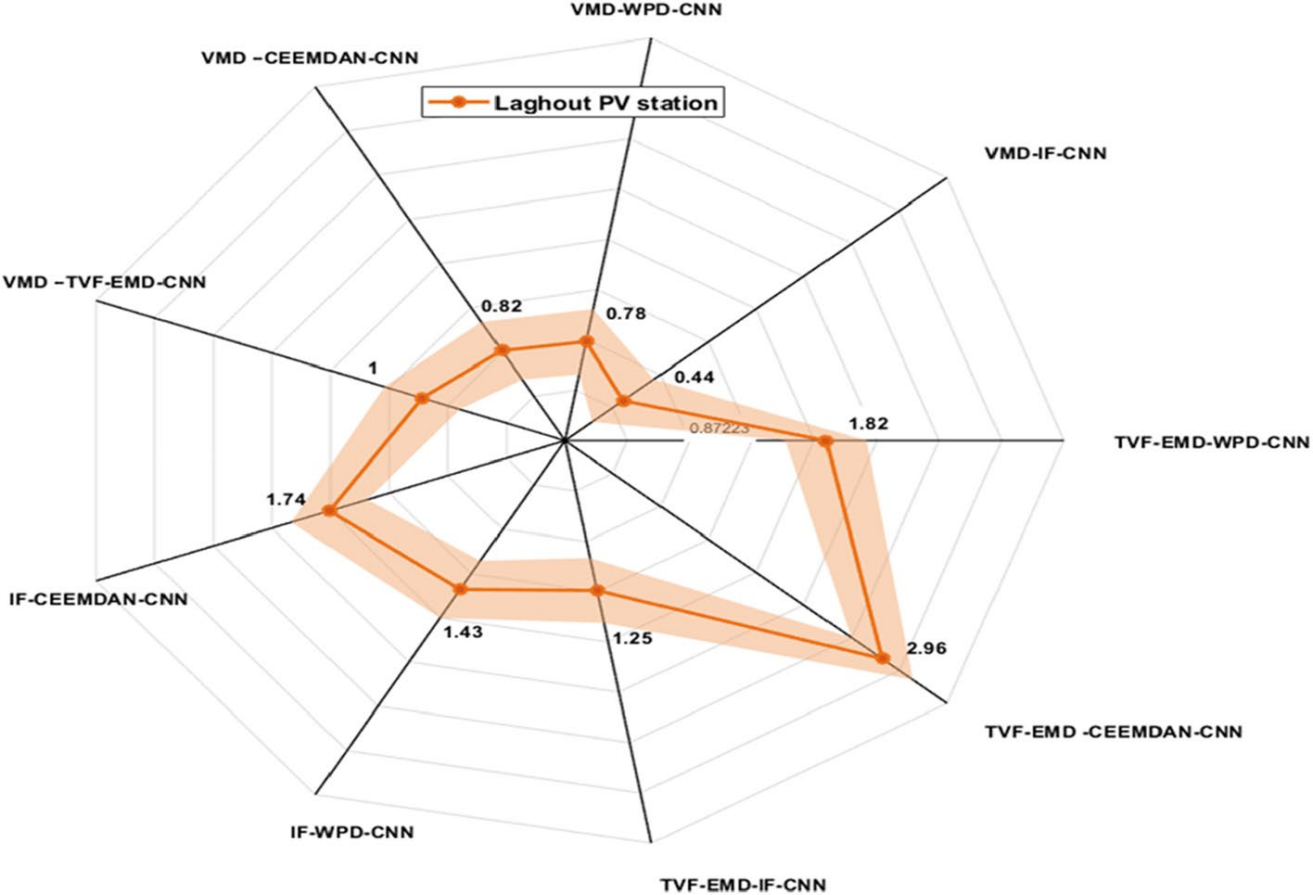
Performance comparison of different decomposition algorithms in terms of nRMSE (Laghouat).

**Figure 11:**
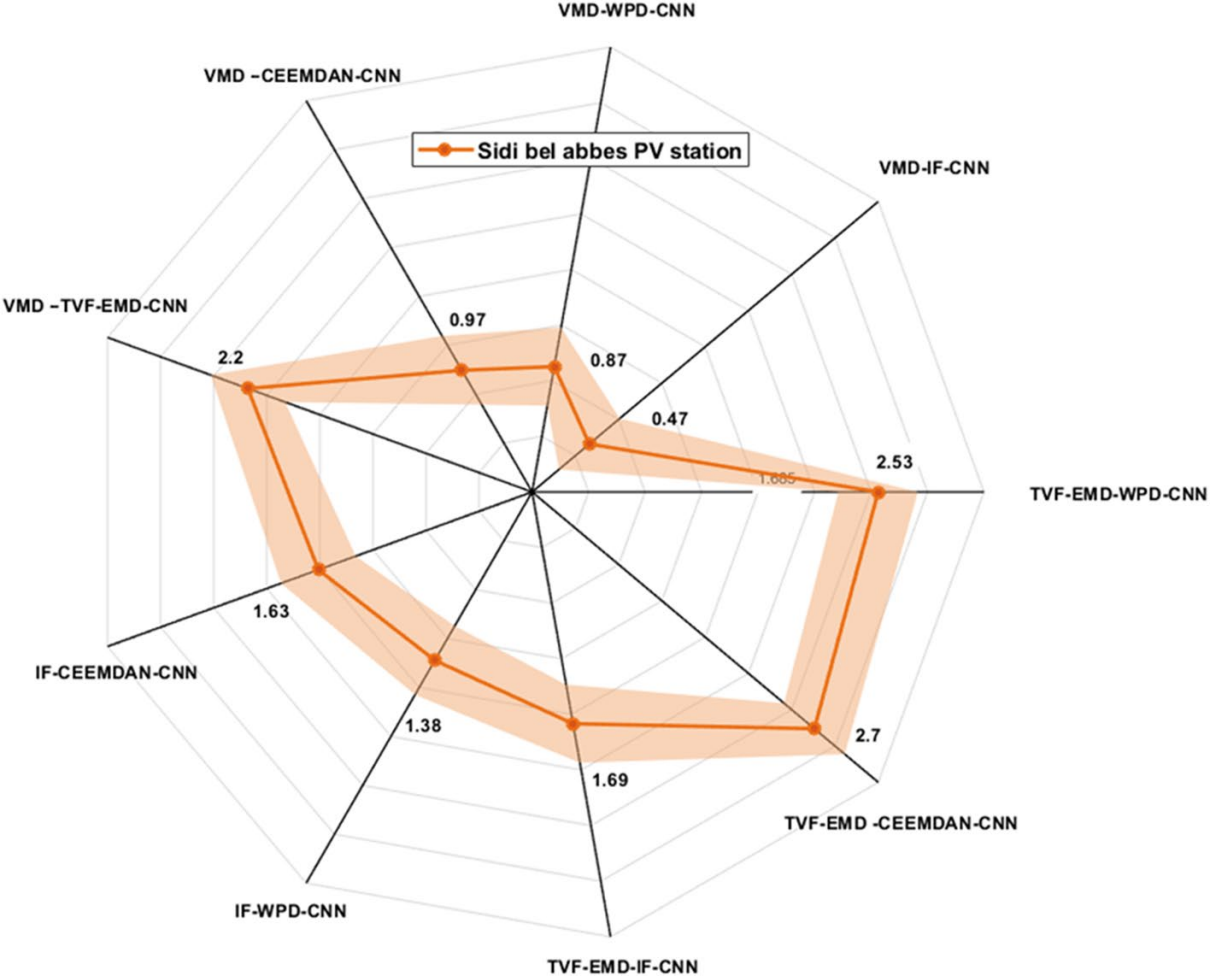
Performance comparison of different decomposition algorithms in terms of nRMSE (Sidi bel abbes).

Figures 9, 10 and 11 exhibits the forecasting outcomes of all suggested multi-scale approach combinations in terms of the nRMSE indicator. According to the findings shown in Fig. 9, all suggested combinations produce strong forecasting results and increase the forecasting precision of individual decomposition approaches; in which the nRMSE error of the proposed hybridization technique varies within the range of [0.44–4].

**Figure 12:**
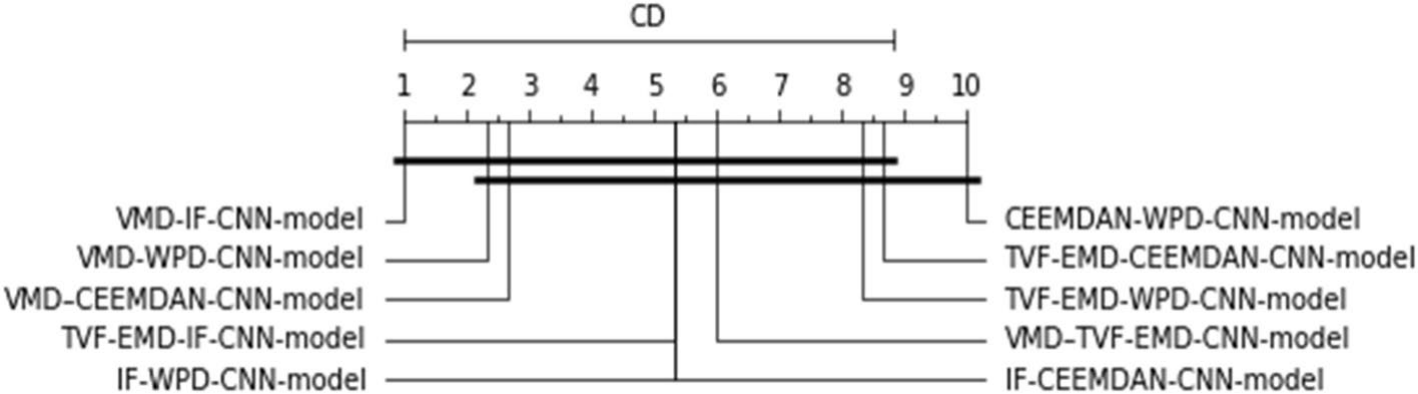
The Friedman-Nemenyi test is applied to all datasets and methodologies.

As can be observed, after the employment of the Friedman–Nemenyi statistical test with 95% confidence, it was discovered that the suggested VMD-IF-CNN-model combination approach delivers the best forecasting performance, and outperforms its counterpart hybrid models for used time series data (See Fig. 12).

Furthermore, an effective PV power-forecasting algorithm should not be time-consuming; however, the suggested forecasting strategy is based on a deep learning technique, which requires time during the training phase. This indicates that training the deep CNN model may take a long time if a large number of similar multi-scale samples are employed. In this respect, Forward Stepwise Regression Algorithm (FSRA) is utilized for selecting the most appropriate IMFs input for PV power forecasting. The primary purpose of identifying the most significant IMFs inputs is to eliminate redundant features, which reduces the computation costs and model complexity^89^. The threshold between approved and rejected features was empirically established during the feature selection process. The developed models are assessed and trained by sequential feature sets obtained by including the next essential IMF elements continuously. The statistical forecasting metrics of the three analyzed PV stations are obtained by two scenarios, the suggested IF-VMD-CNN model with all IMFs elements, and the proposed IF-VMD-FSRA-CNN model combined with a feature selection approach (FSRA), which are illustrated in Table 9 for the three grid-connected photovoltaic plants. The benefit of combining our forecasting technique with a feature selection approach on forecasting accuracy and information space saving is observed. For all analyzed PV power stations, the FSRA algorithm was very useful in minimizing the amount of IMFs inputs necessary for training our deep neural network model while maintaining the forecasting quality of PV power output.

**Table 9:** Improvement of the developed IF-VMD-CNN-model combined with FSRA technique.

Forecasting horizon	Model	RMSE	nRMSE	MABE	nMAE	r (%)	Number of used IMFs components
Laghouat station 30-min	IF-VMD-FSRA-CNN	110.98	0.454	73.592	0.3009	0.999	120
	IF-VMD-CNN	108.07	0.442	73.717	0.3015	0.999	162
Sidi Bel Abbés station 15-min	IF-VMD-FSRA-CNN	22.233	0.498	16.693	0.374	0.999	98
	IF-VMD-CNN	21.288	0.477	16.359	0.366	0.999	144
Ghardaia station 30-min	IF-VMD-FSRA-CNN	8.114	1.594	6.079	1.202	0.999	77
	IF-VMD-CNN	7.870	1.546	5.734	1.133	0.999	161

The undesired IMF features differ slightly by regional location, and forecasting time step, taking the case of the Laghouat PV station 30 min ahead forecasting, the selected IMFs components are 120 IMFs, and the unwanted IMFs numbers are 42. For Sidi Bel Abbés station, the FSRA algorithm removes 46 unneeded IMFs elements from the entire dataset while maintaining the forecasting effectiveness of the IF-VMD-CNN model. The FSRA technique at Ghardaia station removes 84 redundant IMFs elements from the entire dataset while retaining the forecasting ability of the proposed IF-VMD-CNN model. The FSRA algorithm was useful in reducing the amount of IMFs elements for training our model while preserving forecasting effectiveness. This reveals the findings that FS techniques can improve the accuracy of PV power forecasting methods. It is concluded that using a feature selection technique in training the suggested methodology improves forecasting accuracy and computation efficiency. The efficacy of FS methods is highly dependent on the dataset employed since the appropriate quantity of input varies from one PV plant to another. In addition, for each forecasting time step in each analyzed location, a distinct quantity of IMFs was employed as inputs. The findings clearly show that the proposed technique not only provided a remarkable precision rate but also ensured precise forecasting results throughout all periods and regions examined.

An additional evaluation was carried out to compare our proposed model with existing state-of-the-art models in PV power forecasting. The complexity of directly comparing our model to those from previous studies arises from variations in data duration, input variables, climate conditions, and the fact that most studies focus solely on one-step-ahead forecasting at a specific time scale. According to Table 10, our hybridization approach achieves superior forecasting performance relative to numerous preceding efforts. The IF-VMD-FSRA-CNN method we propose demonstrates enhanced suitability for forecasting PV power output, highlighting its efficacy and potential advancements in the field.

**Table 10:** Comparison against the state-of-the-art PV forecasting models.

Authors (year)	Forecasting method	Forecasting horizon	Plan capacity	Data period	Location	Performance metrics
Wang et al.^79^	EEMD-SE-RVM	Hourly scale	30 MW	July 2011 to June 2012	Jiangsu-China	MAPE (%) = 4.05
						FIAW (%) = 0.244
Du et al.^80^	VMD-mRMR-DBN	15–30 min	–	2013	Yunnan, Gansu China	RMSE (MW) = [0.73–0.78]
						MAE (MW) = [0.4–0.44]
						TIC (%) = [0.0053–0.0061]
Zhang et al.^81^	WT-NBDM	15–30 min	20 MW	2014	Gansu-China	MAPE (%) = [4.4–10.6]
						RMSE (MW) = [0.51–1.45]
						MAE (MW) = [0.3–1.05]
Kushwaha et al.^42^	W-RVFL	20 Min	15 kW	2017	Gandhinagar India	MAPE (%) = [3.0–3.6]
						RMSE (kW) = [0.29–0.34]
						R2 = [0.993–0.99]
AlHakeem et al.^82^	WT + GRNN + PSO	1 Hourly	15 kW	–	Ashland-Oregon, United States	MAPE (%) = [8.5–10.4]
						RMSE (MW) = [9.4–11.88]
VanDeventer et al.^83^	GASVM	1 Hourly	3 kW	–	Victoria, Australia	MAPE (%) = 1.7
						RMSE (W) = 11.226
Li et al.^47^	WPD-LSTM	5 Min	25.52 kW	June 1, 2014 to May 31, 2015	Alice Springs, Australia	MAPE (%) = 2.4
						RMSE (kW) = 0.235
Ospina et al.^84^	WT + LSTM-DNN	30 min	12.6 MW		Florida	R = 0.975
						MAPE (%) = [3.55–3.76]
Niu et al.^40^	RF-CEEMD-DIFPSO-BPNN	15 min	800 kW	March 2016–March 2017	Canada	RMSE (kW) = [14.5–17.3]
						MAPE (%) = [10.6–12.4]
Behera et al.^85^	EMD-SCA-ELM	15 Min	11.2 kW	–	Odisha, India	MAPE (%)__15_ = 1.88
		30 Min				MAPE (%)__30_ = 2.52
		60 Min				MAPE (%)__60_ = 3.13
Pan et al.^86^	I-ACO-SVM	5 Min	5.04 kW	2018–2019	Alice Springs, Australia	RMSE (kW) = 0.187
						MAE (kW) = 0.157
						R2 = 0.997
Current research paper	IF-VMD-FSRA-DCNN	30 Min	60 MwP 12 MWp 1.1 MWp	2 years	Laghouat Sidi Bel Abbés Ghardaïa	NRMSE (%)___ = 0.44
		15 Min				NRMSE (%)___ = 0.47
		30 Min				NRMSE (%)___ = 1.54

## Limitations of the study

Our model demonstrates considerable potential in forecasting photovoltaic power yet encounters several challenges. These challenges encompass reliance on the quality of data, a propensity for overfitting when using small datasets, questions about its adaptability to various geographical locations and types of PV systems, and the need for significant computational resources due to its complexity.

In this research, our focus was primarily on the three region, constrained by the availability of data. It is imperative for future studies to broaden their scope to encompass additional regions characterized by diverse weather conditions, especially areas prone to cloudy skies. Examining the efficacy of different PV panel technologies across these varied climates could shed light on the versatility and effectiveness of forecasting models in differing environmental settings.

Moreover, although we employed deep learning techniques such as CNNs, LSTMs, which have shown encouraging outcomes, there remains room for exploration of other sophisticated deep learning architectures. Techniques like the Transformer and Informer, known for their prowess in time series forecasting, present promising avenues for future work. Investigating these models could uncover new strategies to enhance the accuracy and efficiency of PV power forecasting models.

## Conclusion

The present study investigates the performance assessment and short-term forecasting of three PV systems of a 73.1 MW located in Algeria. The proposed forecasting method, VMD-IF-FSRA-CNN is presented to deal with dynamic changes in PV power production 15–30 min ahead. It was tested on three grid-connected PV power plants, and the results were compared to hybrid and stand-alone models. The findings show that the VMD-IF-FSRA-CNN model outperforms the state-of-the-art techniques and exhibits high forecasting accuracy for short-term prediction of PV power. Therefore, this work concentrates on the issue of forecasting PV power in the absence of climatic data, using few historical PV plant data as inputs to the suggested model. to estimate the future PV power output. An in-depth examination of several decomposition approaches coupled with a deep learning algorithm was carried out to get a better insight into what drives the performance of the PV dataset. This study indicates that the proposed combination mechanism is more suitable for multi-site time series forecasting, and could be used for other grid-connected PV power plants.

For future work, we aim to enhance our forecasting models by exploring more sophisticated deep learning methods in conjunction with sky imager data for PV power forecasting. This direction is intended to improve the accuracy and adaptability of our forecasting models by incorporating detailed visual observations of the sky. Such advancements will not only refine solar irradiance predictions but also broaden the models' applicability across diverse geographical locations and environmental conditions. Our ongoing research efforts will focus on developing these integrated models to further optimize the utilization of renewable energy resources, contributing to significant progress in the field of PV power forecasting.

## Data Availability

The datasets used and/or analysed during the current study available from the corresponding author on reasonable request.

## Abbreviations

ACO: Ant colony optimization
ANFIS: Adaptive neuro-fuzzy inference system
ANN: Artificial neural networks
AR: Auto-regression
ARIMA: Auto-regressive integrated moving average
ARMAX: Auto-regressive moving average extrapolation
a-Si: One amorphous
BiGRU: Bi-directional gated recurrent unit
BI-LSTM: Bidirectional long short-term memory
BPNN: Back propagation neural network
CEEMD: Complementary ensemble empirical mode decomposition
CGAN: Conditional generative adversarial model network
CNN: Convolutional neural network
DBN: Deep belief network
DCNN: Deep convolutional neural network
DL: Deep learning
EEMD: Empirical mode decomposition
ELM: Extreme learning machine
EMD: Empirical mode decomposition
FCBF: Fast correlation-based filter
GA: Genetic algorithm
GANs: Generative adversarial model networks
GPR: Gaussian process regression
GRU: Gated recurrent unit
HSV: Hue saturation value
ICEEMDAN: Improved complete ensemble empirical mode decomposition with adaptive
IEA: International energy agency
IEMD: Improved empirical mode decomposition
IF: Iterative filtering
IGIVA: Improved grey ideal value approximation
LR: Linear regression
LS-SVM: Least squares support vector machines
LSTM: Long short-term memory
MAE: Mean absolute error
MAPE: Mean absolute percentage error
MEMD: Multivariate empirical mode decomposition
MLP: Multi-layer perceptron
MOcsa: Multi-objective chameleon swarm algorithm
MODWT: Maximum overlap discrete wavelet transform
MPSO: Modified particle swarm optimization
MRE: Mean relative error
MRMGR: Maximize relevancy minimize global redundancy
m-Si: Eight monocrystalline noise
NWP: Numerical weather prediction
PR: Performance ratio
p-Si: One polycrystalline
PSO: Particle swarm optimization
PV: Photovoltaic
RBF: Radial basis function
RMSE: Root mean square error
RNN: Recurrent neural network
RVFL: Random vector functional link
SARIMA: Seasonal autoregressive integrated moving average
SCA: Sine cosine algorithm
SMAPE: Symmetric mean absolute percentage error
SPVP: Solar photovoltaic plant
SVM: Support vector machine
TSF-CGAN: Time series conditional generative adversarial model network
VAE: Variational autoencoder
VMD: Variational mode decomposition
WGANGP: Wasserstein GAN with gradient penalty
WPD: Wavelet packet decomposition
WT: Wavelet transform
